# Mode 4 restrictiveness and services trade

**DOI:** 10.1007/s10290-022-00481-2

**Published:** 2022-09-24

**Authors:** Anirudh Shingal

**Affiliations:** S.P. Jain Institute of Management & Research, Mumbai and European University Institute, Florence. Address for correspondence: S.P. Jain Institute of Management & Research (SPJIMR), Bhavan’s Campus, Munshi Nagar, Dadabhai Road, Andheri West, Mumbai, 400058 India

**Keywords:** Services trade, Mode 4, Services suppliers, OECD STRI, Gravity model, F1, F10, F13

## Abstract

Despite the importance of services trade and “servicification” of economic activity, “Mode 4” accounted for only 2.9% of total services trade in 2017. While existing literature has estimated services trade costs, effects of barriers to Mode 4 trade have not yet been quantified. We contribute by constructing a composite index to quantify regulatory barriers to the movement of service suppliers, using qualitative information embedded in OECD data on services trade restrictions, and examining its relationship with services trade. Structural gravity estimates suggest that a one standard deviation rise in Mode 4 restrictiveness reduces bilateral services exports by 8%; the adverse effects are even larger for intermediate services exports. Results using aggregate data show that the constructed index is negatively correlated with services imports delivered non-digitally alluding to complementarities between modes of supply and cross-modal “effects”. Moreover, there is considerable heterogeneity in the results across services sectors in both aggregate and bilateral analysis.

## Introduction

Trade in services is important for countries across the world. According to data from the WTO, between 2010 and 2019, trade in commercial services grew by 52% for the G20 and 50% for LDCs, while global trade in commercial services grew by 54%; significantly, exports of commercial services alone witnessed a 108% rise for LDCs over this period. Services are not just an important source of foreign exchange revenue and associated employment and household income, but they are also important for economic growth and development by virtue of their role as inputs into production in all sectors of economic activity (“servicification”). In fact, the share of services in global trade nearly doubles once we account for services trade in value-added terms (WTO, 2019). Moreover, realization of many sustainable development goals (SDGs) depends on the performance of a range of specific services (Fiorini & Hoekman, 2018).

The quality, price and availability of services inputs is determined by a mix of factors, including infrastructure connectivity network investments, the restrictiveness of trade and investment policies for goods and services, and the investment climate/business environment. Empirical evidence suggests that services trade and FDI in services fosters productivity growth by inducing greater competition in domestic markets and providing manufacturing firms access to higher-quality, more varied, and cheaper services inputs, which benefits producers of both goods and services (Arnold et al., 2011, 2016; Beverelli et al., 2017). However, trade costs for services are higher than trade costs for goods, and the rate of decline observed for services trade costs since the early 2000s has been much less than that for goods (Miroudot et al., 2013).

These costs are especially salient for services delivered by the “temporary movement of natural persons” or “Mode 4” trade in WTO GATS parlance^1^, which inter alia explains the low share of Mode 4 trade in total services trade. According to WTO’s Trade in Services by Modes of Supply (TiSMoS) dataset, in 2017^2^, 59.3% of global trade in services was delivered by Mode 3, 27.6% by Mode 1, 10.2% by Mode 2 and only 2.9% by Mode 4. In fact, irrespective of the level of development, the share of Mode 4 in services trade hovers around 3%^3^ though it was even lower at 2.1% in 2005 and 2.5% in 2010.

**Fig. 1:**
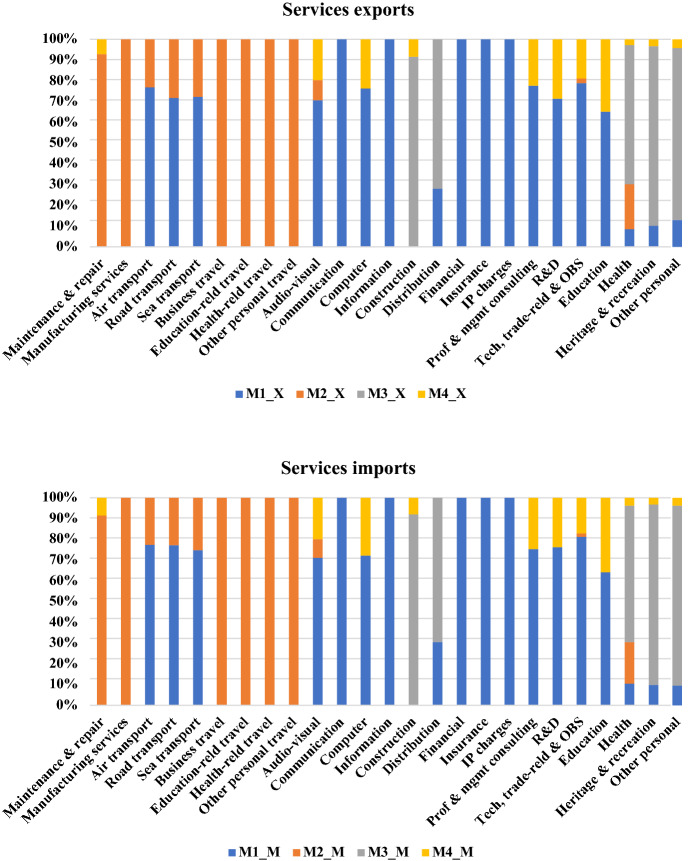
Distribution of services trade by mode of supply and sector (2017). *Source*: WTO TiSMoS; own calculations.* Note*: R&D = Research and development; OBS = Other business services; IP = Intellectual property

Mode 4 may not be used for transacting services trade in all sectors; for instance, WTO TiSMoS data suggest that financial and insurance services are completely delivered cross-border while travel services are wholly delivered by Mode 2. But even in sectors relying on the movement of people, there is significant variation in Mode 4 shares, which suggests the presence of policy impediments. Illustratively, Fig. 1 shows considerable heterogeneity in the modal distribution of services trade by sector in the year 2017. Services were delivered by Mode 4 in only 11 of the 25 sectors reported in Fig. 1, but there was significant variation in Mode 4 shares even in these sectors. The Mode 4 dominant sectors include education, computer, other business and audio-visual services; in contrast, Mode 4 shares were much lower in maintenance & repair, construction, health and personal services.^4^

In general, barriers to services trade do not take the form of border measures such as tariffs, but are rather embedded in regulatory frameworks. However, barriers to Mode 4 trade also include border measures such as visas, work permits and quotas and are therefore more distinct. At the same time, labour market tests for work permits for service providers, and nationality/citizenship/permanent residency requirements for license to practice are examples of “behind-the-border” regulatory barriers constraining Mode 4 trade.

Trade costs for services, for intermediate vs final services, and for disaggregated services sectors, have been computed “top-down” by Miroudot et al. (2013) and Miroudot and Shepherd (2016) using the theory-based methodology of Novy (2013) as well as estimated in a structural gravity framework (WTO, 2019). Measures of regulatory impediments to services trade - the services trade restrictiveness indices (STRI) put together independently by the World Bank and the OECD - have also been used to examine the effects of regulatory incidence and heterogeneity on services trade, investment, integration into global value chains, and the membership and depth of of preferential trade agreements (Kox & Nordås, 2007, 2009; Nordås, 2016; Miroudot & Cadestin, 2017; Nordås & Rouzet, 2017; Rouzet & Spinelli, 2016; Rouzet et al., 2017; Andrenelli et al., 2018; Shingal et al., 2018; Benz & Jaax, 2020; Egger & Shingal, 2021). However, the effect of barriers specific to Mode 4 services trade has not yet been quantified.

Against this background, we contribute by constructing a composite index^5^ to quantify regulatory barriers to the movement of service suppliers, using qualitative information from the OECD’s STRI data, and examining its relationship with services trade using both aggregate and bilateral data. Note that the OECD’s STRI database also provides non-numeric, including Yes/No, responses to regulatory measures that affect Mode 4 trade. We convert these responses to numeric values and use them to construct a composite index (see Sect. 3 for details) that can be used in empirical analysis as an alternative to the quantitative data on “restrictions to movement of people” available in the OECD STRI database. Relative to the OECD data, we add value by also quantifying the individual regulatory measures underlying the OECD STRI data on “restrictions to movement of people” to enable more granular analysis (see Table 2 for details) and construct both simple- and weighted-average indices.

Estimates based on a structural gravity model of bilateral services trade suggest that a one standard deviation rise in importer-specific Mode 4 restrictiveness reduces exports of intermediate services by 10.3% but those of final services by only 0.2%. Thus, barriers to the movement of services suppliers seem to have a significantly pronounced adverse effect on the cross-border flow of intermediate services, which has negative implications for other sectors of economic activity given the servicification narrative. The adverse effects of exporter-specific Mode 4 restrictions are slightly more pronounced for both intermediate and final services. Meanwhile, sector-level analysis displays considerable heterogeneity in the impact of barriers to Mode 4 trade, with financial and business services affected the most, followed by construction, transport and distribution services.

Results from aggregate analysis show that our constructed index is negatively correlated with services imports in three of the four modes of services delivery - Modes 2 through 4 - that require proximity between buyers and sellers. Notably, countries and sectors reliant on these Modes, which accounted for over 70% of global services trade in 2017 according to TiSMoS data, have already been more adversely affected by the COVID-19 pandemic (Shingal, 2021). Moreover, the global regulatory environment, especially that governing commercial presence, may have become more restrictive for services covered by the OECD STRI database in 2020 (OECD, 2021). In such a scenario, any enhancement of existing regulatory restrictions on the movement of people is likely to further exacerbate services trade costs (for instance see (Benz et al., 2020)) and be even more detrimental to post-pandemic economic recovery.

Our aggregate results also confirm complementarities between different ways in which services trade is transacted. In particular, doubling Mode 4 restrictiveness at the mean is found to be associated with a 50% decline in Mode 4 services imports on average and a 41.6% and 35% decline in services imports delivered by Modes 2 and 3, respectively. Sector-level analysis suggests that the overall results may be driven by other business; personal, cultural and recreational; and maintenance & repair services.

The rest of the paper is structured as follows. Section 2 provides a brief review of the growing literature on the effects of services regulation and trade barriers. Section 3 describes the construction of the Mode 4 restrictiveness index. Section 4 discusses the empirical models used to examine the relationship between the constructed index and services trade. Section 5 describes the data and its sources while Sect. 6 presents and discusses results from estimation. Section 7 concludes.

## Related literature: effects of services regulation and trade barriers

Services regulatory measures affect international trade and investment in services by increasing both the fixed cost of entering a market and the variable cost of servicing it. The importance and potentially trade- and investment-inhibiting impact of domestic regulation on service sector performance has received some attention in the literature (for instance see (Kox & Nordås, 2007, 2009; Nordås, 2016)). Regulatory heterogeneity has also been shown to exert a significantly negative impact on bilateral services trade delivered via commercial presence (Kox & Nordås, 2009; Nordås, 2016). In fact, regulatory heterogeneity has been found to account for 21 percent of total trade costs in services along with trade policy barriers (WTO, 2019). Regulatory incidence and heterogeneity have also been shown to be significant determinants of countries’ propensities to negotiate preferential services trade agreements (Egger & Shingal, 2021) and of their deeper commitments in such agreements relative to their WTO GATS commitments (Shingal et al., 2018).

Barriers to trade in services have been found to adversely affect trade, investment and value-chain integration, including at the firm level. Rouzet and Spinelli (2016) find regulatory restrictions in broadcasting, construction, storage, and air and maritime transport sectors to enable firms in these sectors to charge higher mark-ups, pointing to the potential for pro-competitive gains from regulatory liberalization. Nordås and Rouzet (2017) find higher regulatory restrictiveness to be associated with lower imports in the importing country across several sectors including legal services, telecommunications, commercial banking, insurance, maritime transport and courier services. Rouzet et al. (2017) find services firms’ exports at both the extensive and intensive margin to be inversely related with regulatory restrictions in the importing jurisdictions. Benz and Jaax (2020) provide ad-valorem equivalents of the OECD STRI on cross-border trade in five services sectors - business, communications, financial, insurance and transport services - and find these policy-induced trade costs to be high on average, despite services trade having tripled in value over the last two decades. Miroudot and Cadestin (2017) find larger services-trade restrictiveness to be inversely related with bilateral flows of service value-added within GVCs. Andrenelli et al. (2018) and Backer and Miroudot (2018) show how the restrictiveness of trade and investment in services sectors affects production of MNEs that use such services for organizing their value chains besides influencing their export versus FDI decision in accessing foreign markets. Data restrictiveness has also been associated with adverse effects both on the productivity of domestic firms (Ferracane and van der Marel, 2018) and on imports of services (Ferracane & Marel) in countries imposing data-restrictive policies.

Thus, while there is a growing literature studying the impact of services trade restrictions along different dimensions, the effects of barriers specific to Mode 4 trade have not yet been quantified. This paper aims to bridge this gap, thereby complementing the analysis in Benz et al. (2020), who examine the impact of regulatory restrictions - implemented on health and safety grounds following the COVID-19 outbreak in March 2020 - on the cross-border movement of people on services trade costs.^6^

## Constructing the Mode 4 restrictiveness index

We construct a composite index of regulatory measures constraining Mode 4 trade as an alternative to the quantitative data on “restrictions to movement of people” available in the OECD STRI database. The OECD’s database on these restrictions also includes qualitative information on 27 measures (across 29 sectors and sub-sectors) of which 24 measures include a “Yes/No” answer and the remaining three^7^ measures include quantitative information. While this information is now available for the years 2014 to 2020, we restrict our period of analysis to 2014-2017 as services trade data by mode of supply, used in our aggregate analysis, are only available until 2017 in the TiSMoS database. Details on the coverage of countries, sectors and STRI measures are included in Annex A and Tables 1 and 2, respectively.

In constructing the index, we first convert the Yes/No response to 24 of the 27 STRI measures into binary quantitative values where N=0 and Y=1^8^, such that the values range from 0 (least) to 1 (most) restrictive. For the three remaining measures that include data on limitations on the duration of stay for services providers, we convert these data into a quantitative index with values lying between 0 and 1 as follows:^9^

1 $$\begin{aligned} dur\_index_{jkt}\;=\;\frac{Dur^{Max}-Dur_{jkt}}{Dur^{Max}} \end{aligned}$$

where $Dur_{jkt}$ is the duration (in number of months) in sector *k* in country *j* in year *t* and $Dur^{Max}$ is the maximum duration of stay for services providers in any sector across countries; this “global” maximum of 61 months is the maximum time period considered “temporary” in the OECD STRI database. The numerator of Eq. 1 thus measures the “gap” to “best practice” (amongst the 45 countries for which these data are available) at the sector-level such that the larger the gap, the more restrictive is the country imposing the measure. The ratio in Eq. 1 ensures that the values of the “duration index” lie between 0 and 1. We thus “convert” the information embedded in all 27 STRI measures into numeric values.

We then compute sector-specific simple averages, $R\_index_{jkt}^{s}$, of these numeric values for each country and year. However, these simple averages do not reflect the existence of binding measures i.e. the fact that in some sectors, certain combinations of a few measures completely close the sector for trade^10^. Following Geloso et al. (2015), we identify eight such sector-specific STRI measures^11^ as binding and in each case, we replace the value of $R\_index_{jkt}^{s}$ by 1, indicating maximum restrictiveness. Taking the simple average of the resultant $r\_index_{jkt}^{s}$ over all sectors then yields the simple average Mode 4 restrictiveness index, $r\_index_{jt}^{s}$, for each country in each year.

Since simple averages mask sectoral differences, we also use weighted averages to construct the aggregate index where the weights are the country’s sectoral shares in its total services import value in an earlier time period^12^. Thus:

2 $$\begin{aligned} r\_index_{jt}^{w}\;=\;\frac{{\scriptstyle k}\sum r\_index_{jkt}^{s}*\left( \frac{M_{jkt-10}}{M_{jt-10}}\right) \frac{}{}}{{\scriptstyle k}\sum \left( \frac{M_{jkt-10}}{M_{jt-10}}\right) } \end{aligned}$$

where $r\_index_{jt}^{w}$ is the aggregate weighted average index for country *j* at time *t*; $r\_index_{jkt}^{s}$ is the simple average index for country *j* at time *t* (each year from 2014 to 2017) at sector-level *k*; $M_{jkt-10}$ is country *j*’s import value in sector *k* at time $t-10$ (each year from 2005 to 2008); and $M_{jt-10}$ is country *j*’s total services import value at time $t-10$ (each year from 2005 to 2008).^13^

## Empirical strategy

The constructed index captures regulatory restrictions on the movement of service providers in the implementing jurisdiction. We assess the relationship between the index and services trade using both bilateral and aggregate data.

### Bilateral analysis

The bilateral analysis is based on a structural gravity model. Following Anderson and van Wincoop (2003), the gravity model takes the following form:

3 $$\begin{aligned} X_{ijt}\;=\;\frac{E_{jt}Y_{it}}{Y_{t}}\left( \frac{\phi _{ijt}}{P_{jt}\Pi _{it}}\right) ^{1-\sigma } \end{aligned}$$

where $X_{ijt}$ is the value of nominal bilateral exports of services between origin *i* and destination *j* at time *t*, $E_{j}$ is the expenditure on services in the destination market from all origins, $Y_{i}$ is the sale of services at destination prices from *i* to all destinations, *Y* is world output of services at delivered prices, $\tau {}_{ij}$ are the bilateral trade costs, $\sigma$ is the elasticity of substitution amongst services and $P_{j},$ $\Pi {}_{i}$ are the (inward and outward, respectively) multilateral resistance terms (MRTs) as defined in this literature.

Trade costs in $\phi _{ijt}$ arise from sources such as geographical distance between trading partners $[ln(DIST_{ij})]$; cultural distance proxied by dummy variables identifying whether the trading partners share a common border ($CNTG_{ij}$), had a colonial relationship ($CLNY_{ij}$), and share a common language $(LANG_{ij})$; and membership of preferential trade agreements ($PTA_{ijt}$).

Recent advancements in the estimation of structural gravity models advocate the use of three-way fixed effects to mitigate endogeneity-induced biases in estimation (for instance see (Baier & Bergstrand, 2007; Baier et al., 2014; Piermartini & Yotov, 2016)). The dyadic trade cost variables ($lnDIST_{ij}$, $CNTG_{ij}$, $CLNY_{ij}$ and $LANG_{ij}$) are thus subsumed in bilateral pair-wise fixed effects ($\alpha _{ij}$), leading to the following equation:

4 $$\begin{aligned} X_{ijt}\;=\;exp[\beta _{1}PTA{}_{ijt}+\alpha _{ij}+\mu _{it}+\gamma _{jt}]+\epsilon _{ijt} \end{aligned}$$

where $\mu _{it}$ and $\gamma _{jt}$ are the time-varying exporter and importer fixed effects that proxy the outward and inward MRTs, respectively, and $\varepsilon {}_{ijt}$ is the error term.

The dependent variable in Eq. 4 also includes data on intra-national services trade flows, which not only makes the model theory-consistent (Fally, 2015) but also enables us to quantify the effect of non-discriminatory trade barriers such as our constructed Mode 4 restrictiveness index, $r\_index_{jt}^{w}$ (which is otherwise collinear with the time-varying importer fixed effects), using an interaction term between the constructed index and a binary dummy ($INTL_{ij}$) that takes the value one for international trade flows and the value zero for intra-national trade flows (see Anderson et al. (2018); Benz and Jaax (2020) for similar applications). Since Mode 4 barriers in the exporting country can also have an adverse effect on services exports^14^, we further include the interaction between $INTL_{ij}$ and the exporter-specific Mode 4 restrictiveness index, $r\_index_{it}^{w}$, in both distinct and combined specifications. The final combined bilateral estimating equation takes the following form:

5 $$\begin{aligned} X_{ijt}\;=\;exp[\beta _{1}r\_index_{it}^{w}.INTL_{ij}+\beta _{2}r\_index_{jt}^{w}.INTL_{ij}+\beta _{3}PTA{}_{ijt}+\alpha _{ij}+\mu _{it}+\gamma _{jt}]+\epsilon _{ijt} \end{aligned}$$

Equation 5 is estimated using the Poisson Pseudo-Maximum Likelihood (PPML; (Silva & Tenreyro, 2006)) using data on cross-border and intra-national trade flows in services sectors^15^ from the EORA26 MRIO database (Lenzen et al., 2012, 2013). The use of the PPML also accounts for heteroskedasticity-related concerns in estimation and is now the preferred choice for estimating structural gravity models (Piermartini & Yotov, 2016). Note that we prefer using the weighted indices ($r\_index_{it}^{w}$, $r\_index_{jt}^{w}$) in our main specifications as they account for the relative importance of individual sectors in services trade. This said, we also consider the simple average indices in our sensitivity analysis. We expect estimated $\beta _{1},$ $\beta _{2}$ to be negative and estimated $\beta _{3}$ to be positive.

Finally, since bilateral services trade data are also available at the sector-level, we also include disaggregated analysis to examine the effect of the exporter- and importer-specific Mode 4 restrictiveness indices, both aggregate and sector-specific, on bilateral trade in individual services sectors. Note that the sector definitions in EORA are more aggregated than those in TiSMoS, so the bilateral analysis using the weighted indices is based on ten broad sectors (reported in Table 4, panel A), while that using the sector-specific indices is only possible for four broad sectors (see Table 4, panel B). Moreover, the EORA database provides information on services trade flows catering to both intermediate and final demand, which enables us to examine the effects along this dimension as well.

### Aggregate analysis

Since bilateral services trade data are not available by mode of supply, we also use data from WTO TiSMoS that allows us to examine the relationship between the constructed index and services imports by mode of supply. We do so by estimating the following import equation using fixed effects specifications:

6 $$\begin{aligned} lnM_{jt}^{m}\;=\;\alpha r\_index_{jt}^{w}+\beta _{\mathbf {z}}Z{}_{\mathbf {z}jt}+\delta _{j}+\delta _{t}+\varepsilon _{jt} \end{aligned}$$

where $M_{jt}^{m}$ is the services imports of country *j* in year *t* delivered by Mode *m*; $r\_index_{jt}^{w}$ is the constructed aggregate weighted average Mode 4 restrictiveness index; $Z_{\mathbf {z}jt}$ is a vector of country-time varying controls; $\delta _{j}$ and $\delta _{t}$ are country and year fixed effects; and $\varepsilon _{jt}$ is the error term. Again, we prefer using the weighted index in our main specification as it accounts for the relative importance of individual sectors in services trade but also consider the simple average index in our sensitivity analysis.

The empirical specification and choice of explanatory variables are motivated in existing literature (Cali & te Velde, 2011; Martínez-Zarzoso et al., 2017; Hoekman & Shingal, 2020). The control vector, $Z_{zjt}$, comprises a measure of country size - the log of population ($POP_{jt}$); a measure of geographic distance to global markets - the log of market penetration ($MP_{jt}$) computed as a distance ($d_{ij}$) weighted measure of other countries’ GDP ($GDP_{it}$) i.e. $MP_{jt}\;=\;\sum _{i}(GDP_{it}/d_{ij})$; a measure of domestic prices - log of the consumer price index ($CPI_{jt}$); a measure of government effectiveness ($GE_{jt}$) to reflect institutional strength; and the log of inward foreign direct investment ($FDI_{jt}$). We expect each of these variables to be positively correlated with services imports by mode of supply, justifying their choice as control variables.

While explicitly focusing on Mode 4 restrictions, we also control for all other trade costs affecting services trade via the inward multilateral resistance (IMR) term as defined in Anderson and van Wincoop (2003). The IMR terms are constructed following Larch and Yotov (2016), using estimates of the time-varying importer fixed effects obtained from the structural gravity model of bilateral services trade in Eq. 4.

Finally, since TiSMoS data are also available at the sector-level, we again include disaggregated analysis to examine the effect of Mode 4 restrictiveness, both aggregate and sector-specific, on total imports of individual services sectors by mode of supply. While the availability of sectors in TiSMoS is very disaggregated (see Fig. 1), the results from aggregate analysis using the weighted and sector-specific indices only report those sector-Mode combinations in Tables 6 and 7 where (i) services trade was transacted over 2014-17 as reported in the TiSMoS database and (ii) the estimated coefficients on the restrictiveness indices were statistically significant at conventional levels.

## Data sources and description

Since the Mode 4 restrictiveness index is constructed for 45 countries in the OECD’s STRI database over 2014-2017, the dependent and control variables span the same country and time period. Bilateral data on cross-border and intra-national trade flows in services sectors come from the EORA26 MRIO database (Lenzen et al., 2012, 2013). Services trade data (with the world as a partner) by mode of supply are sourced from WTO TiSMoS. The control variables are sourced as follows: the consumer price index ($CPI_{jt}$), foreign direct investment ($FDI_{jt}$) and population ($POP_{jt}$) are taken from the World Bank’s World Development Indicators (WDI); market penetration ($MP_{jt}$) is computed using bilateral distance data from CEPII (Head et al., 2010) and GDP data from the WDI; government effectiveness ($GE_{jt}$) is sourced from the Worldwide Governance Indicators (Kaufmann et al., 2011). The PTA membership dummy ($PTA_{ijt}$) is constructed using data from the WTO RTA-IS database, for services agreements notified under Article V of the GATS.

The empirical analysis is carried out on 45, primarily OECD, countries over 2014-2017, leading to a sample of 180 observations in the aggregate analysis and over 16,000 observations in the bilateral analysis. Summary statistics are reported in Annex Table 1.

**Fig. 2:**
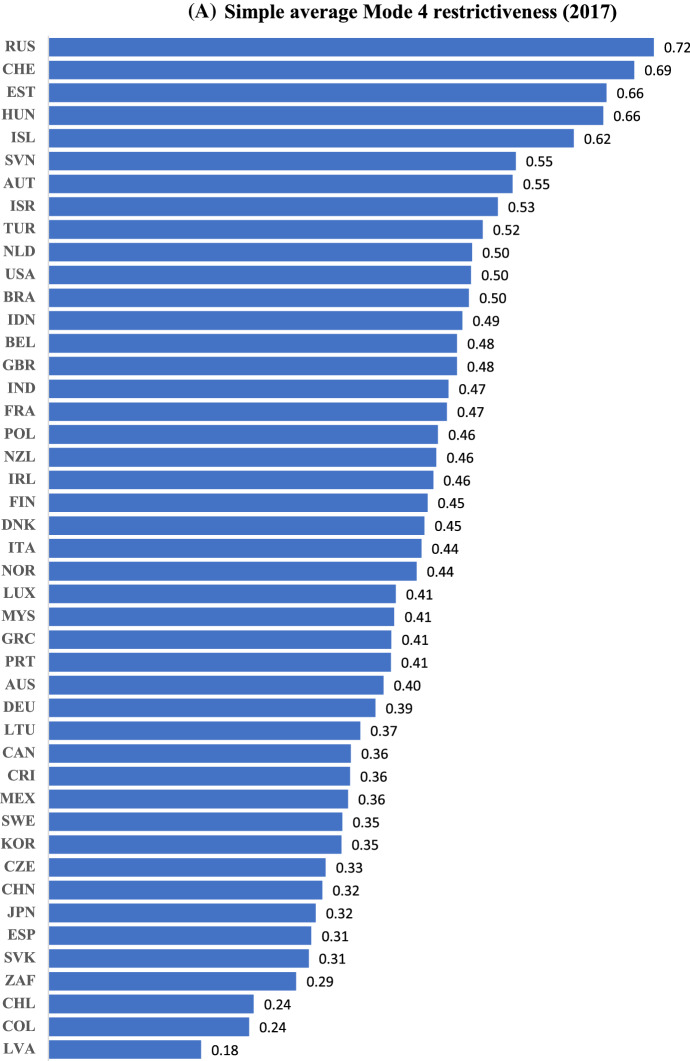

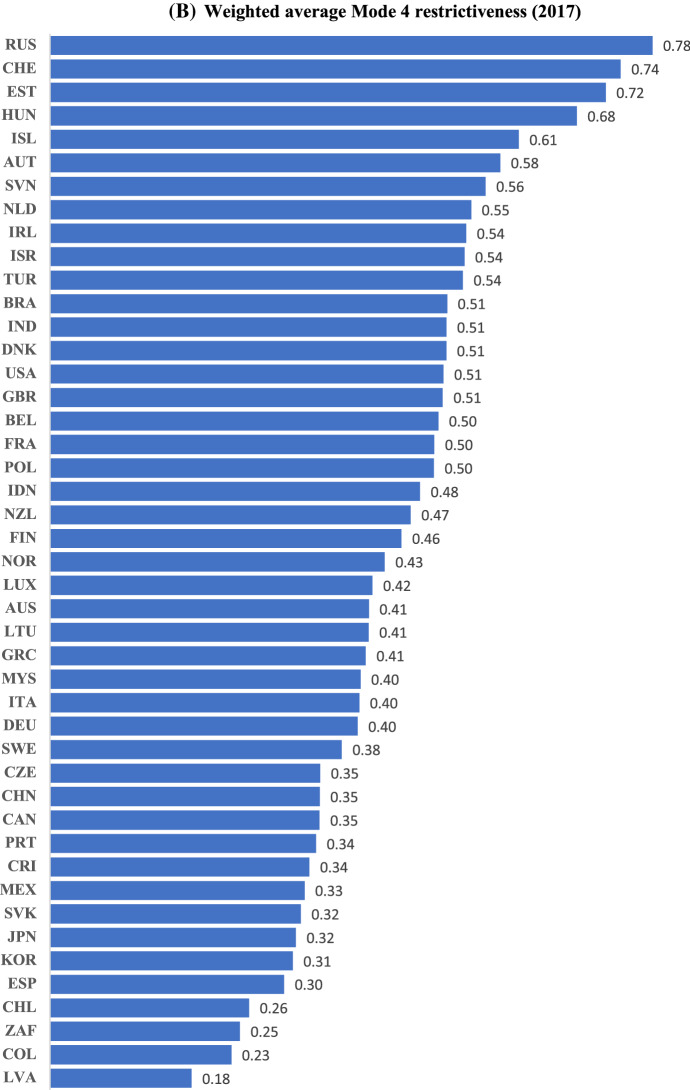
**A** Simple average Mode 4 restrictiveness (2017). *Source*: OECD STRI; own calculations.* Note*: The aggregate index by country is constructed using simple averages of the constructed index across sectors. ** B** Weighted average Mode 4 restrictiveness (2017). *Source*: OECD STRI; own calculations.* Note*: The aggregate index by country is constructed using weighted averages of the constructed index across sectors, where the weights are sectoral shares in total services imports by value in an earlier time period for each country

Figure 2A and B present the average Mode 4 restrictiveness in 2017 based on simple and weighted averages, respectively. The two distributions are broadly similar; the average scores range from 0.18 for Latvia (at the bottom end of the distribution) to 0.72/0.78 for Russia (at the top end). The average score for non-OECD countries (0.4) is found to be lower than that for the OECD (0.45/.46) as Latvia, Colombia, and South Africa are amongst the least restrictive countries in the sample while nine of the top ten most Mode 4 restrictive countries (barring Russia at the top) belong to the OECD.

Correlation between our simple-average Mode 4 restrictiveness index and OECD STRI data on “restrictions to movement of people” is high with a coefficient exceeding 0.8; Latvia, South Africa and Spain remain amongst the least restrictive countries to Mode 4 trade and Estonia, Switzerland and Iceland amongst the most restrictive. While our overall empirical findings are thus likely to be broadly similar using the OECD data, the use of our index enables more granular analysis such as that provided in Table 2 below. Additionally, we construct both simple- and weighted-average indices, while the OECD data are not trade-weighted.

Table 1 reports the average Mode 4 restrictiveness score by sector in 2017 averaged over all sample countries and the count of countries for which the (simple) average score at the sector-level was more than the sectoral mean^16^. The most Mode 4 restrictive sectors include auditing, legal, architecture, engineering and insurance. In contrast, computer, audio-visual, courier and logistics services were amongst the least restrictive, which seeems to support these sectors being amongst the more Mode 4 dominant services trading sectors in Fig. 1.

**Table 1:** Count of countries for which the simple average Mode 4 restrictiveness by sector exceeds the sectoral mean (2017). *Source*: OECD STRI; own calculations

Sector	Sectoral average	Count of countries
Accounting and auditing services	0.449	21
Accounting services	0.543	37
Air transport	0.455	22
Architecture services	0.644	33
Audiovisual - Broadcasting	0.436	25
Audiovisual - Motion pictures	0.461	22
Audiovisual - Sound recording	0.396	25
Auditing services	0.726	43
Commercial banking	0.455	23
Computer services	0.395	25
Construction - Engineering	0.457	21
Construction services	0.455	22
Courier services	0.396	25
Distribution services	0.453	22
Engineering services	0.586	27
Insurance	0.514	20
Insurance - Actuaries	0.422	16
Insurance - Broking and agency services	0.583	19
Legal services	0.622	29
Legal services - Domestic law	0.690	41
Legal services - International law	0.352	26
Logistics cargo-handling	0.453	22
Logistics customs brokerage	0.544	21
Logistics freight forwarding	0.453	22
Logistics storage and warehouse	0.453	22
Maritime transport	0.486	18
Rail freight transport	0.503	18
Road freight transport	0.485	18

Table 2 reports the average Mode 4 restrictiveness score by STRI measure in 2017, again averaged over all sample countries, and the count of countries for which the (simple) average score by measure exceeded the mean. The most Mode 4 restrictive measures include absence of a temporary licensing system; and nationality/residency/domicile requirements for practice. In contrast, the least Mode 4 restrictive measure were laws or regulations that establish a process for recognizing qualifications gained abroad and the requirement to practise locally for 1 year or take local exams.

**Table 2:** Count of countries for which the simple average Mode 4 restrictiveness by STRI measure exceeds the STRI measure mean (2017). *Source*: OECD STRI; own calculations

STRI measure	Average by STRI measure	Count of countries
A temporary licensing system is in place	0.719	24
Appointed actuaries must be nationals or residents	0.529	16
At least one engineer must be licensed for the issuance of construction permits	0.337	21
Domicile required for Licence to practice	0.492	23
Foreign construction engineers are required to practice locally for at least 1 year	0.337	21
Foreign construction engineers are required to take a local examination	0.337	21
Foreign professionals are required to practice locally for at least 1 year	0.381	25
Foreign professionals are required to take a local examination	0.381	25
Foreign providers have to completely re-do the university degree, practice and exam in the domestic country	0.384	19
Labour market tests: contractual services suppliers	0.439	22
Labour market tests: independent services suppliers	0.439	22
Labour market tests: intra-corporate transferees	0.439	22
Laws or regulations establish a process for recognising qualifications gained abroad	0.423	25
Laws or regulations establish a process for recognising qualifications in engineering gained abroad	0.337	21
Limitation on duration of stay for contractual services suppliers (months)	0.439	22
Limitation on duration of stay for independent services suppliers (months)	0.439	22
Limitation on duration of stay for intra-corporate transferees (months)	0.439	22
Membership in the professional association is closed to foreigners	0.422	16
Memo: Licence or authorisation is required to practice	0.400	23
Nationality or citizenship required for construction engineers	0.440	20
Nationality or citizenship required for Licence to practice	0.368	21
Other restrictions to movement of people	0.439	22
Prior or permanent residency is required for Licence to practice	0.428	21
Quotas: contractual services suppliers	0.439	22
Quotas: independent services suppliers	0.439	22
Quotas: intra-corporate transferees	0.439	22
Residency is required to practice	0.493	21

## Results from estimation

### Bilateral analysis

#### Total bilateral services exports

The results from estimating Eq. 5 on bilateral services exports - intermediate^17^, final^18^ and total - are reported in Table 3; standard errors are clustered by dyad-year in each case. The coefficient estimates reported in columns (1) and (2) suggest that a one standard deviation rise in the exporter- and importer-specific weighted-average Mode 4 restrictiveness indices reduces bilateral intermediate services exports by 10.5% and 10.3%^19^, respectively. In contrast, the 0.3% and 0.2% reduction in bilateral final services exports from a one standard deviation increase in exporter- and importer-specific Mode 4 restrictiveness is much smaller (see columns 4 and 5); for total services in columns (7) and (8), the corresponding change is −7.9% and −7.8%, respectively. When the two interaction terms are included together in columns (3), (6) and (9), the importer-specific term is dropped due to collinearity.

The significantly pronounced adverse effect of barriers to the movement of services suppliers on cross-border flow of intermediate services also has negative implications for other sectors of economic activity given the servicification narrative. Interestingly, except for column (6), PTA-membership does not have a statistically significant positive effect on bilateral services exports of the 45 sample countries in these results, for intermediate, final or total services. Note that 24 of the 45 sample countries are EU Member States where the Internal Market for Services has been a major achievement but the time span of our analysis means that any “EU services effect” is subsumed in the pairwise fixed effects. Similarly, the little variation in the PTA variable over the short time span of analysis and heterogeneity in the “PTA-effect” across sectors (see Table 4) likely account for the absence of a statistically significant positive effect in these results.

**Table 3:** Mode 4 restrictiveness reduces total bilateral services exports (PPML estimates)

	(1)	(2)	(3)	(4)	(5)	(6)	(7)	(8)	(9)
VARIABLES	**X**$^{\mathbf {INT}}_{\mathbf {ijt}}$			**X**$^{\mathbf {FNL}}_{\mathbf {ijt}}$			**X**$^{\mathbf {TOT}}_{\mathbf {ijt}}$		
PTA$_{ijt}$	0.006	0.015	0.009	0.000	0.000	0.025***	0.009	0.013	0.010
	(0.016)	(0.017)	(0.020)	(0.000)	(0.000)	(0.004)	(0.007)	(0.009)	(0.010)
r_index$^{w}_{it}$*INTL$_{ij}$	−0.907***		−0.921***	−0.024***		0.032	−0.677***		−0.692***
	(0.120)		(0.123)	(0.003)		(0.033)	(0.101)		(0.104)
r_index$^{w}_{jt}$*INTL$_{ij}$		−0.892***			−0.017***			−0.670***	
		(0.120)			(0.002)			(0.102)	
Observations	16,470	16,470	4,050	16,470	16,470	4,050	16,470	16,470	4,050
Pseudo-R2	1.000	1.000	1.000	1.000	1.000	1.000	1.000	1.000	1.000
*Fixed effects:*									
Exporter-Importer	YES	YES	YES	YES	YES	YES	YES	YES	YES
Importer-Year	YES	YES	YES	YES	YES	YES	YES	YES	YES
Exporter-Year	YES	YES	YES	YES	YES	YES	YES	YES	YES
% change from 1 s.d. rise in restrictiveness	−10.5	−10.3	−10.6	-0.3	−0.2		−7.9	−7.8	−8.1

In columns (3), (6) and (9), interaction terms of INTL $_{ij}$ with both r_index $^{w}_{it}$ and r_index $^{w}_{jt}$were used, but the latter term was dropped in estimation due to collinearity. Robust standard errors, clustered by dyad-year, included in parentheses in all specifications. Levels of significance: *10%, **5%, ***1 %

#### Sector-level bilateral services exports

Sector-level information on bilateral services exports in the EORA database also enables an examination of the effects of Mode 4 restrictiveness, using both the aggregate weighted-average index and the sectoral simple-averaged indices. While the former analysis can be undertaken for ten^20^ services sectors for which information on bilateral exports is available from the EORA database, the latter analysis is confined to four broad sectors that correspond to the construction of the sectoral Mode 4 restrictiveness indices - construction, distribution (retail and wholesale trade), financial and business services, and transport services. The results from these analyses are reported in Table 4, panels A and B, respectively. While panel A reports the results for total bilateral services exports for each of the ten sectors, panel B does so for intermediate and total bilateral services exports for each of the four broad sectors. The standard errors are clustered by dyad-year in each case.

**Table 4:** Mode 4 restrictiveness reduces sector-level bilateral services exports (PPML estimates)

	(1)	(2)	(3)	(4)	(5)	(6)	(7)	(8)	(9)	(10)
VARIABLES	**X**$^{\mathbf {Const}}_{\mathbf {ijt}}$	**X**$^{\mathbf {Dist}}_{\mathbf {ijt}}$	**X**$^{\mathbf {Edu \& Hlth}}_{\mathbf {ijt}}$	**X**$^{\mathbf {Fin \& Bus}}_{\mathbf {ijt}}$	**X**$^{\mathbf {Hotels}}_{\mathbf {ijt}}$	**X**$^{\mathbf {Main \& Rep}}_{\mathbf {ijt}}$	**X**$^{\mathbf {Post \& Tel}}_{\mathbf {ijt}}$	**X**$^{\mathbf {PubAd}}_{\mathbf {ijt}}$	**X**$^{\mathbf {Recycle}}_{\mathbf {ijt}}$	**X**$^{\mathbf {Trans}}_{\mathbf {ijt}}$
*A: Using constructed aggregate weighted-average index*										
Exporter-specific Mode 4 restrictiveness										
PTA$_{ijt}$	0.020	0.056***	0.000	0.006	−0.001	0.012	−0.024***	−0.094**	0.011	0.002
	(0.014)	(0.010)	(0.003)	(0.015)	(0.004)	(0.008)	(0.003)	(0.037)	(0.010)	(0.004)
r_index$^{w}_{it}$*INTL$_{ij}$	−0.550***	−0.683***	−0.577***	−0.749***	0.502	−0.739***	−0.869***	−0.556***	−0.003	−0.588***
	(0.099)	(0.107)	(0.041)	(0.112)	(0.357)	(0.089)	(0.125)	(0.098)	(0.204)	(0.107)
Observations	16,470	16,470	16,470	16,470	16,470	16,470	16,470	16,470	16,104	16,470
Pseudo-R2	1.000	1.000	1.000	1.000	1.000	1.000	1.000	1.000	1.000	1.000
% change from 1 s.d. rise in restrictiveness	−6.5	−8.0	−6.8	−8.7		−8.6	−10.0	−6.6		−6.9
Importer-specific Mode 4 restrictiveness										
PTA$_{ijt}$	0.026**	0.064***	0.004	0.008	-0.004	0.023***	−0.023***	−0.102***	0.016	0.004
	(0.012)	(0.009)	(0.003)	(0.014)	(0.005)	(0.008)	(0.002)	(0.034)	(0.017)	(0.006)
r_index$^{w}_{jt}$*INTL$_{ij}$	−0.544***	−0.683***	−0.577***	−0.735***	0.073	−0.654***	−0.874***	−0.549***	−0.079	−0.607***
	(0.092)	(0.108)	(0.042)	(0.113)	(0.234)	(0.081)	(0.123)	(0.091)	(0.155)	(0.105)
Observations	16,470	16,470	16,470	16,470	16,470	16,470	16,470	16,470	16,380	16,470
Pseudo-R2	1.000	1.000	1.000	1.000	1.000	1.000	1.000	1.000	1.000	1.000
%* change from 1 s.d. rise in restrictiveness*	−6.4	−8.0	−6.8	−8.6		−7.7	−10.1	−6.5		−7.1

VARIABLES	(1)	(2)	(3)	(4)	(5)	(6)	(7)	(8)	
	Finance & business		Transport		Construction		Distribution		
	X$^{\mathbf {Int}}_{\mathbf {ijt}}$	X$^{\mathbf {Tot}}_{\mathbf {ijt}}$	X$^{\mathbf {Int}}_{\mathbf {ijt}}$	X$^{\mathbf {Tot}}_{\mathbf {ijt}}$	X$^{\mathbf {Int}}_{\mathbf {ijt}}$	X$^{\mathbf {Tot}}_{\mathbf {ijt}}$	X$^{\mathbf {Int}}_{\mathbf {ijt}}$	X$^{\mathbf {Tot}}_{\mathbf {ijt}}$	
*B: Using constructed sectoral simple-average indices*									
Exporter-specific Mode 4 restrictiveness									
PTA$_{ijt}$	−0.014	0.005	−0.020*	0.002	−0.121***	0.020	0.026**	0.056***	
	(0.019)	(0.014)	(0.011)	(0.004)	(0.028)	(0.014)	(0.012)	(0.010)	
r_index$^{s}_{ikt}$*INTL$_{ij}$	−2.345***	−1.894***	−0.803***	−0.634***	−1.091***	−0.706***	−0.573***	−0.572***	
	(0.181)	(0.156)	(0.107)	(0.091)	(0.156)	(0.136)	(0.096)	(0.087)	
Observations	16,470	16,470	16,470	16,470	16,470	16,470	16,470	16,470	
Pseudo-R2	1.000	1.000	1.000	1.000	1.000	1.000	1.000	1.000	
%* change from 1 s.d. rise in restrictiveness*	−27.0	−22.5	−10.2	−8.2	−13.6	−9.1	−7.4	−7.4	
Importer-specific Mode 4 restrictiveness									
PTA$_{ijt}$	−0.010	0.006	−0.014	0.004	0.045**	0.025**	0.045***	0.063***	
	(0.019)	(0.014)	(0.014)	(0.006)	(0.022)	(0.012)	(0.012)	(0.009)	
r_index$^{s}_{jkt}$*INTL$_{ij}$	−2.316***	−1.867***	−0.811***	−0.646***	−1.070***	−0.703***	−0.575***	−0.571***	
	(0.184)	(0.158)	(0.106)	(0.090)	(0.143)	(0.125)	(0.096)	(0.087)	
Observations	16,470	16,470	16,470	16,470	16,470	16,470	16,470	16,470	
Pseudo-R2	1.000	1.000	1.000	1.000	1.000	1.000	1.000	1.000	
% * change from 1 s.d. rise in restrictiveness*	−26.8	−22.2	−10.3	−8.3	−13.4	−9.0	−7.4	−7.4	

The dependent variable is the level of total bilateral exports in each sector. Robust standard errors, clustered by dyad-year, included in parentheses in all specifications. All specifications include exporter-year, importer-year and bilateral fixed effects. Levels of significance: *10%, **5%, ***1%

There is considerable heterogeneity across sectors in the impact of barriers to Mode 4 trade in the results reported in panels A and B. Irrespective of the source of restrictiveness, the adverse effects are the most pronounced for post and telecommunications, finance and business, distribution, maintenance and repair, and transport services (Table 4, panel A). These findings also show that barriers to services suppliers have negative spillovers in sectors such as eduction and health, and post and telecommunications, which are not directly covered by the underlying data, as well as in sectors such as finance and distribution where Mode 4 is not dominant (see Fig. 1), thereby suggesting complementarities in trade between services sectors and modes of delivery and cross-modal effects. At the same time, these findings show that restrictions such as quotas, economic needs tests, and limitations on duration of stay apply to all sectors, including education and health. Meanwhile, as would be expected, barriers to Mode 4 trade do not have a statistically significant effect on either hotels and restaurants or recycling services. Interestingly, PTA-membership seems to have a statistically significant positive effect on bilateral exports of maintenance and repair, construction and distribution services in these results, ranging from 2.3% to 6.6%^21^.

Turning to the sectoral simple-averaged indices (Table 4, panel B), their adverse effects are the most pronounced for financial and business services, followed by construction, transport and distribution services; again irrespective of the source of restrictiveness. PTA-membership also seems to have a statistically significant positive effect on bilateral exports of construction and distribution services in these results, ranging from 4.6% each for intermediate services to 2.5% and 6.5% for total services.

### Aggregate analysis

#### Total aggregate services imports by mode of supply

Table 5 reports the results from the OLS estimation of Eq. 6 for services imports by each mode of supply, with standard errors clustered by country-year in each case.^22^

**Table 5:** Relationship between the weighted Mode 4 restrictiveness index and aggregate services imports (OLS estimates)

	(1)	(2)	(3)	(4)
VARIABLES	ln(M$^{\mathbf {M1}}_{\mathbf {jt}}$**)**	ln(M$^{\mathbf {M2}}_{\mathbf {jt}}$**)**	ln(M$^{\mathbf {M3}}_{\mathbf {jt}}$**)**	ln(M$^{\mathbf {M4}}_{\mathbf {jt}}$**)**
r_index$^{w}_{jt}$	−0.1785	−0.4157**	−0.3504**	−0.5003**
	(0.1162)	(0.2082)	(0.1634)	(0.2892)
ln(POP$$_{jt})$$	0.5829	−0.2664	−0.9411	0.3187
	(0.6270)	(1.0066)	(0.8330)	(1.6029)
ln(MP$$_{jt})$$	−0.0017	0.0061*	0.0011	−0.0046
	(0.0026)	(0.0034)	(0.0031)	(0.0058)
ln(FDI$$_{jt})$$	0.0078	0.0133	−0.0066	0.0171
	(0.0073)	(0.0134)	(0.0105)	(0.0160)
ln(CPI$$_{jt})$$	−0.8643***	−1.0969**	−0.4961	−1.1237
	(0.3037)	(0.4892)	(0.4199)	(0.6893)
IMR$_{jt}$	0.3873	−4.2754	−6.0021	−1.1557
	(2.7288)	(5.1190)	(4.5625)	(6.5027)
GE$_{jt}$	−0.0730	−0.0470	0.1356	−0.1830
	(0.0961)	(0.1332)	(0.1264)	(0.2208)
Observations	152	152	152	152
R2	0.9987	0.9967	0.9976	0.9944

All estimations include country and year fixed effects. Robust standard errors, clustered by country-year, included in parentheses. Levels of significance: *10%, **5%, ***1%

The Mode 4 restrictiveness index is found to be negatively associated with imports of services delivered by Modes 2-4; the estimated coefficient for Mode 1 services imports is found to be statistically indifferent from zero. Given that the index captures regulatory barriers to the movement of service providers, one would expect the estimated elasticity to be the largest for Mode 4 imports. Encouragingly, this is what we find: a unit increase in Mode 4 restrictiveness (equivalent to more than doubling the value of the index at the mean) is associated with a 50% decline in services imports delivered by the movement of service providers in these results, ceteris paribus and on average^23^. Given that the STRI measures listed in Table 2 also include labour market tests, quotas and limitations on duration of stay for CSSs, ISSs and ICTs, one would also expect the magnitude of the estimated coefficients to be large for Mode 2 and 3 services imports. This is also found to be the case: a unit increase in Mode 4 restrictiveness is associated with a 41.6% and 35% decline in services imports delivered by Modes 2 and 3, respectively, ceteris paribus and on average.

These findings also confirm complementarities between different ways in which services trade is transacted. They also illustrate how barriers in one mode of service delivery can affect another. Such complementarities are obvious, for instance, when establishing commercial presence abroad (Mode 3 trade) leads to intra-corporate transfers (Mode 4 trade) from the home country to the host country. In such a scenario, any restrictions on the movement of ICTs is also likely to have an adverse effect on foreign affiliate transactions. Similarly, a short-duration professional visit abroad (Mode 4 trade) can also generate an appetite for exploring a new country as a tourist (Mode 2 trade), possibly with family. Thus, any curbs on the movements of CSSs and ISSs could also result in a decline in tourism.

Finally, while the R-squared values are close to 1 across specifications in the results reported in Table 5, the estimates of only the consumer price index and the market potential variable report statistical significance, which suggests that the fixed effects capture most of the variation in the dependent variable at the aggregate level.

#### Sector-level analysis using the aggregate weighted average index

The WTO TiSMoS database also includes services trade data by mode of supply for individual services sectors. Since the Mode 4 restrictiveness index is aggregated across sectors by construction, we do not expect sectoral imports to show much correlation with it. Even so, replicating the analysis using the aggregate weighted index at the sector level shows that the overall results for Modes 2-4 in Table 5 may be driven by other business; personal, cultural and recreational; and maintenance & repair services; respectively. Note that the magnitudes of the estimated coefficients on sectoral imports in Table 6 are much larger than those on total imports in Table 5.

**Table 6:** Relationship between the aggregate weighted Mode 4 restrictiveness index and sectoral services imports (OLS estimates)

	(1)	(2)	(3)	(4)
VARIABLES	ln(M$^{\mathbf {PCR\_M3}}_{\mathbf {jt}}$**)**	ln(M$^{\mathbf {Sea\_trans\_M1}}_{\mathbf {jt}}$**)**	ln(M$^{\mathbf {M \& R\_M4}}_{\mathbf {jt}}$**)**	ln(M$^{\mathbf {OBS\_M2}}_{\mathbf {jt}}$**)**
r_index$^{w}_{jt}$	−3.9464***	−0.9487**	−1.2677*	−1.2992*
	(1.3998)	(0.4342)	(0.7031)	(0.7698)
ln(POP$$_{jt})$$	−6.1720	1.3111	−8.1682*	−9.0586
	(4.0092)	(1.2536)	(4.6196)	(6.1427)
ln(MP$$_{jt})$$	−0.0215	−0.0010	0.0258**	−0.0148
	(0.0220)	(0.0048)	(0.0127)	(0.0234)
ln(FDI$$_{jt})$$	−0.0483	−0.0175	0.0213	0.1850*
	(0.1187)	(0.0176)	(0.0542)	(0.1032)
ln(CPI$$_{jt})$$	3.2927	−0.9931**	0.3859	2.8144
	(2.0231)	(0.4346)	(1.2088)	(1.9288)
IMR$_{jt}$	−65.0408*	−6.1103	17.9991	1.8530
	(34.3717)	(5.0670)	(27.9456)	(32.0580)
GE$_{jt}$	0.1025	0.0641	−0.6151	0.3702
	(0.7926)	(0.1339)	(0.3753)	(0.6579)
Observations	152	152	135	152
R2	0.9912	0.9975	0.9835	0.9645

All estimations include country and year fixed effects. Robust standard errors, clustered by country-year, included in parentheses. Levels of significance: *10%, **5%, ***1%. PCR $=$ Personal, cultural and recreational services; M & R $=$ Maintenance and repair services; OBS $=$ Other business services

The estimated coefficients on the aggregate Mode 4 restrictiveness index were found to be statistically indifferent from zero for all other sector-Mode combinations. Moreover, unlike the results reported in Table 5, more control variables exhibit statistical significance now across sectors, especially for maintenance and repair services, though the negative coefficient on the population variable is counter-intuitive, while the IMR term displays the expected negative relationship in the case of personal, cultural and recreational services imports.

#### Sector-level analysis using sector-level restrictiveness indices

We next assess the relationship between Mode 4 restrictiveness indices constructed at the sector-level and sector-level imports for the sectors where such an empirical analysis is possible.^24^ The results from this analysis are reported in Table 7 and suggest negative correlations between sectoral Mode 4 restrictiveness and Mode 3 construction imports and land and maritime transport services delivered via Mode 1.^25^ The large magnitude of the estimated coefficient in the case of construction services delivered via commercial presence likely reflects the presence of STRI measures specific to construction and engineering services in Table 1 as well as Mode 4 barriers on ICTs. Similarly, the moderately high Mode 4 restrictiveness in maritime transport services (see Table 1) and the fact that over 70% of services imports in that sector was transacted via Mode 1 in 2017 (see Fig. 1) translates into the large coefficient estimate observed in that sector in the results reported in Table 7, also confirming the Table 6 findings that barriers to the movement of services suppliers can also harm cross-border services trade^26^.

**Table 7:** Relationship between sectoral Mode 4 restrictiveness indices and sectoral services imports (OLS estimates)

	(1)	(2)	(3)
VARIABLES	ln(M$^{\mathbf {Constrn\_M3}}_{\mathbf {jt}}$**)**	ln(M$^{\mathbf {Sea\_trans\_M1}}_{\mathbf {jt}}$**)**	ln(M$^{\mathbf {Land\_trans\_M1}}_{\mathbf {jt}}$**)**
r_index$^{s}_{jkt}$	−1.1284**	−1.6752***	−0.4098**
	(0.4813)	(0.4764)	(0.2018)
ln(POP$$_{jt})$$	6.8601***	4.8441**	−1.5665
	(1.0041)	(1.9797)	(1.7907)
ln(MP$$_{jt})$$	−0.0169***	0.0096	−0.0049
	(0.0037)	(0.0074)	(0.0044)
ln(FDI$$_{jt})$$	−0.0087	−0.0304	0.0398**
	(0.0205)	(0.0234)	(0.0159)
ln(CPI$$_{jt})$$	0.4948	−1.4300***	0.1663
	(0.4520)	(0.3845)	(0.4770)
IMR$_{jt}$	−3.3409	−18.5594**	−13.2677***
	(3.6071)	(7.5935)	(2.9759)
GE$_{jt}$	−0.1083	0.3444**	0.2923**
	(0.1232)	(0.1515)	(0.1177)
Observations	706	382	780
R2	0.9984	0.9968	0.9974

All estimations include country and year fixed effects. Robust standard errors, clustered by country-year, included in parentheses. Levels of significance: *10%, **5%, ***1%. Constrn $=$Construction services; Sea-trans $=$ Sea-transport services; Land-trans $=$ Land transport services.

Finally, again unlike the aggregate results reported in Table 5, more control variables are found to be statistically significant in sector-level analysis, especially the IMR term, which again displays the expected negative relationship in the case of maritime and land transport services imports.

#### Relationship between Mode 4 restrictiveness and services exports

Given complementarities between services exports and imports, we also replicated the analysis above using both aggregate and sector-level data on services exports by modes of supply. However, the relationship between Mode 4 restrictiveness and services exports was found to be statistically insignificant across sectors and modes of supply. It is possible that such a relationship is more likely observed in services value-added trade data and not in the gross services trade data that the WTO TiSMoS database covers; unfortunately, services value-added trade data are yet not available by mode of supply.

### Sensitivity analysis

#### Replicating analyses using the aggregate simple average index

As a robustness check, we replicated the analyses in Sects. 6.1.1 and 6.2.1 using the aggregate simple-averaged index. The results from both bilateral and aggregate analyses were qualitatively similar to the respective baseline results and are available upon request.

#### Using an alternative estimator in aggregate analysis

Given heteroskedasticity-related concerns in estimation, we also replicated all aggregate analyses in Sect. 6.2 using the PPML. The results from using the PPML were found to be qualitatively similar to those from using OLS and are available upon request.

## Conclusions

Despite the importance of services trade and servicification of economic activity, Mode 4 accounted for less than 3% of total services trade in 2017. Our bilateral analysis, which also mitigates endogeneity-related concerns in estimation, suggests that a one standard deviation rise in Mode 4 restrictiveness reduces bilateral services exports by 8%. Moreover, regulatory restrictions on the movement of services suppliers are found to be particularly harmful for trade in intermediate services, which is likely to have adverse spill-overs effects on the rest of the economy given the servicification narrative. Regulatory barriers to Mode 4 trade are also found to be negatively correlated with services imports in precisely those modes of supply that are already more adversely affected by COVID-19, suggesting that any enhancement of existing regulatory restrictions on such trade is likely to further exacerbate service trade costs and be even more detrimental to post-pandemic economic recovery.

One limitation of the analyses on offer is that bilateral services trade data are not yet available by mode of supply. Moreover, TiSMoS, the source of aggregate services trade data by mode of supply, being a constructed database, relies on fixed shares for most countries, assuming that Mode 4 corresponds to 25% of total balance-of-payments services trade in a majority of sectors (Wettstein et al., 2017). While this may have implications for our aggregate analysis, it is a challenge that can, unfortunately, not be circumvented. This said, as shown in the introduction, there is considerable heterogeneity in the share of Mode 4 in total services trade across countries and sectors in TiSMoS to justify using that database and to making our aggregate analysis more valid. Moreover, the results from our bilateral analysis are broadly consistent with the findings from our aggregate analysis, which further assuages any data quality-related concerns associated with the use of TiSMoS.

Finally, in constructing the indices, we take the OECD STRI data at face value and trust that the measures only/mostly capture restrictions as they apply to Mode 4 trade. We thus abstract from the possibility that some of the “restrictions to movement of people” may also affect other modes of supply or that some of the “sector-specific” measures may also have an impact in other sectors. Indeed, such data quality-related issues may have had a bearing on our results, especially those that suggest cross-modal or cross-sectoral complementarities, but addressing such challenges is beyond the scope of this work. Also note that by definition, Mode 4 involves the temporary movement of natural persons to deliver a service internationally, hence measures affecting permanent migration would not be covered by the STRI database and are therefore not a part of our analysis, even though the positive impact of long-term migration in facilitating Mode 4 trade via diaspora linkages and knowledge of institutional barriers in the host country is well recognized in the gravity-diaspora literature.

## Annex A: List of countries covered by the OECD STRI data

Australia, Austria, Belgium, Brazil, Canada, Chile, China, Colombia, Costa Rica, Czech Republic, Denmark, Estonia, Finland, France, Germany, Greece, Hungary, Iceland, India, Indonesia, Ireland, Israel, Italy, Japan, Korea, Latvia, Lithuania, Luxembourg, Malaysia, Mexico, Netherlands, New Zealand, Norway, Poland, Portugal, Russia, Slovak Republic, Slovenia, South Africa, Spain, Sweden, Switzerland, Turkey, United Kingdom, and United States. (see Table 8)

**Table 8:** Data sources and summary statistics

Variable	Source	Obs	Mean	Std. Dev.	Min	Max
ln(X$$^{INT}_{ijt})$$	EORA	16,470	8.60	2.87	3.30	22.85
ln(X$$^{FNL}_{ijt})$$	EORA	16,470	6.93	3.32	1.69	23.34
ln(X$$_{ijt})$$	EORA	16,470	8.86	2.96	3.49	23.82
ln(M$$^{M1})$$	WTO, TiSMoS	180	10.27	1.36	7.32	13.02
ln(M$$^{M2})$$	WTO, TiSMoS	180	9.22	1.33	6.03	12.12
ln(M$$^{M3})$$	WTO, TiSMoS	180	10.98	1.37	7.88	14.05
ln(M$$^{M4})$$	WTO, TiSMoS	180	7.84	1.56	3.92	10.55
r_index$^{s}$	(Simple average) OECD, STRI	180	0.43	0.11	0.18	0.72
r_index$^{wt}$	(Weighted) OECD, STRI	180	0.44	0.13	0.18	0.78
ln(Pop)	World Bank, WDI	180	3.00	1.81	-1.12	7.23
ln(MP)	World Bank, WDI; Head et al. (2010)	166	17.18	2.53	7.75	23.06
ln(CPI)	World Bank, WDI	180	4.74	0.11	4.58	5.16
ln(FDI)	World Bank, WDI	165	9.56	1.58	5.81	13.14
GE	Kaufmann et al. (2011)	180	1.06	0.66	−0.29	2.11

The index is constructed using qualitative information on “Restrictions to movement of people” from the OECD Services Trade Restrictiveness Index (STRI) Regulatory Database. TiSMoS $=$ Trade in Services by Mode of Supply; WDI =World Development Indicators; MP$_{jt}$ is computed using bilateral distance data from CEPII (Head et al., 2010) and GDP data from the WDI; GE$_{jt}$ is sourced from the Worldwide Governance Indicators (Kaufmann et al., 2011)

## Footnotes

1 There are four different ways in which services are traded internationally: Mode 1 (“cross-border services trade”) that includes the entire range of services transacted via the internet e.g. online medical transcription services; Mode 2 (“consumption abroad” where the buyer travels overseas to consume a service e.g. tourism); Mode 3 (“commercial presence” by a foreign affiliate in the domestic economy and the affiliate’s transactions e.g. international retail banking services); and Mode 4 (“movement of natural persons” where the seller travels abroad to deliver a service e.g. IT professionals working onsite abroad and intra-corporate transferees).

2 This is the latest year for which TiSMoS provides services trade data disaggregated by modes of supply.

3 While the average Mode 4 share (2.9%) is the same for the group of upper-middle income countries, it is higher for the group of high income (3.1%) and lower-middle income countries (3.2%) but lower for the group of low-income countries (2.7%), according to the Word Bank’s income classification of countries.

4 Mode 4 accounted for 36 percent of the total import of education services; about a quarter of imports of computer and other business services; and a fifth of audio-visual services imports. The sectoral distribution of Mode 4 was similar for services exports. Meanwhile, maintenance & repair; manufacturing services and travel were almost completely delivered by Mode 2, while construction, distribution and personal services were largely delivered by Mode 3. Services trade in the remaining sectors was largely transacted cross-border.

5 While our “bottom up” approach explicitly focuses on regulatory restrictions, we also control for all other trade costs affecting services trade in the estimating equations in our aggregate analysis via the multilateral resistance term (see Sect. 4 for details).

6 Benz and Jaax (2020) hypothesize scenarios in which countries close their borders to passengers but leave freight trade open, resulting in the associated STRIs becoming more restrictive and estimate the affected services trade costs to increase by an average of 12% of export values across sectors and countries in the medium term.

7 These measures pertain to limitations (in number of months) on duration of stay for (i) contractual service suppliers (CSSs); (ii) independent service suppliers (ISSs); and (iii) intra-corporate transferees (ICTs).

8 Note that two measures in the STRI data relate to laws or regulations that establish a process for recognizing qualifications gained abroad and another measure relates to the existence of a temporary licensing system. These measures support Mode 4 trade and have thus been reverse-coded (i.e. N=1 and Y=0) in constructing the index.

9 There is considerable heterogeneity in the duration of stay across countries, on average, ranging from only 1 year for Switzerland, Costa Rica, Finland, Israel and Turkey (across service professionals) to 4 years in the case of Australia and Denmark (but only 2 years for ISSs); 4–5 years for the UK; and 5 years in the case of China, Japan, Latvia (but only 1 year for ISSs) and South Africa (though only 4 years for ICTs). The sample maxima point to the ground that the remaining countries in the STRI database can cover in extending the duration of stay to these services professionals (if not eliminating limitations on these stay durations altogether), thereby greatly reducing costs imposed on Mode 4 trade.

10 For instance, if nationality or citizenship is required to deliver a service then that market is effectively closed even if there are no other restrictions on Mode 4 trade. We would like to thank an anonymous referee for pointing this out and motivating the resulting change in strategy deployed in constructing the Mode 4 restrictiveness index.

11 These include “A temporary licensing system is in place”; “Appointed actuaries must be nationals or residents”; “Domicile required for Licence to practice”; “Foreign providers have to completely re-do the university degree, practice and exam in the domestic country”; “Nationality or citizenship required for Licence to practice”; “Nationality or citizenship required for construction engineers”; “Prior or permanent residency is required for Licence to practice”; and “Residency is required to practice”. These binding measures apply to eleven sectors in the OECD STRI database: Accounting services; Architecture services; Auditing services; Engineering services; Legal services; Insurance; Legal services - Domestic law; Legal services - International law; Insurance - Broking and agency services; Logistics customs brokerage; and Construction - Engineering.

12 We weight the Mode 4 restrictiveness index constructed over 2014-17 using country-specific sectoral shares in total services imports varying annually over 2005-08 i.e. a decade before each year of analysis and before the global financial crisis.

13 Note that the construction of $r\_index_{jt}^{w}$ is based on 14 data-source-dependent services sectors that are common between the OECD STRI and the WTO TiSMoS databases. These sectors include audio-visual, air transport, architecture, communications, computer-related, construction, distribution, engineering, financial, insurance, legal & accounting, post & courier, sea transport and road transport services.

14 We would like to thank an anonymous referee for this suggestion.

15 These include construction, distribution, education and health, finance and business, hotels and restaurants, maintenance and repair, post and telecommunications, public administration, recycling and transport services.

16 While the number of Mode 4-specific STRI measures varies significantly across sectors, a comparison of average restrictiveness across sectors is feasible as we average the restrictiveness across STRI measures in constructing sector-specific indices.

17 These cover services exports used as intermediate inputs in production across sectors in the destination country.

18 These cover services exports meeting final private and government consumption and investment demand in the destination country.

19 These are calculated as $[e^{(Coefficient^{\hat{AB}}*s.d.^{A}*Mean^{B})}-1]*100$ for an interaction term AB.

20 These include construction, distribution, education and health, finance and business, hotels and restaurants, maintenance and repair, post and telecommunications, public administration, recycling and transport services.

21 This is calculated as $(e^{\hat{\beta _{3}}}-1)*100$.

22 We experimented with GMM specifications in the aggregate analysis to control for potential endogeneity in the Mode 4 restrictiveness-services import relationship but these results lacked statistical significance. We also used index values (both simple and weighted averaged) for the year 2014 as instruments in IV regressions using 2017 data on the sample of 45 countries. Interestingly, the exogeneity of the relationship between Mode 4 imports and the Mode 4 restrictiveness index was not rejected in the diagnostic statistics, but the IV estimates lacked statistical signifiance for all modes of supply. We thus refrain from attributing any causality to the findings from our aggregate analysis; those results are best expressed as conditional correlations.

23 In the log-linear model specified in Eq. 6, the approximate % change in the dependent variable from a unit increase in the explanatory variable is given by $100*\hat{\alpha }$.

24 This includes the following sectors: accounting & legal services; architechture; audio-visual; computer; construction; courier; distribution; engineering; financial; insurance; and transport services. But Table 7 only reports those sector-Mode combinations where the estimated coefficient on $r\_index_{jkt}^{s}$ was statistically significant at conventional levels.

25 We also used index values for the year 2014 as instruments in IV regressions using 2017 data on a larger country-sector sample than in the sector-level analysis using the weighted-average index. The IV estimates lacked statistical signifiance for all sectors and modes of supply, with the exception of computer services imports delivered by Mode 1. While this result was also weakly significant at the 10% level, it suggests that an increase in Mode 4 restrictiveness has an adverse effect on Mode 1 computer services imports. This again confirms complementarities between ways in which business is conducted in this sector. All IV results are available upon request.

26 Indeed, cross-border trade depends on truck drivers, ship crews and airline crews being able to cross borders. This became blatantly clear at the outset of the COVID-19 crisis and was a major factor behind supply chain disruptions.
